# Prospectively predicting BPaMZ phase IIb/III trial outcomes using a translational mouse-to-human platform

**DOI:** 10.1128/aac.00615-24

**Published:** 2024-09-17

**Authors:** Janice J. N. Goh, Qianwen Wang, Nan Zhang, Niurys de Castro Suarez, Annamarie E. Bustion, Eric L. Nuermberger, Rada Savic

**Affiliations:** 1 Department of Bioengineering and Therapeutic Sciences, University of California, San Francisco, San Francisco, California, USA; 2 Center for Tuberculosis Research, Department of Medicine, Johns Hopkins University School of Medicine, Baltimore, Maryland, USA; St. George's, University of London, London, United Kingdom

**Keywords:** *Mycobacterium tuberculosis*, PKPD, preclinical translation, clinical trial prediction, mechanistic model, drug regimens

## Abstract

Despite known treatments, tuberculosis (TB) remains the world’s top infectious killer, highlighting the pressing need for new drug regimens. To prioritize the most efficacious drugs for clinical testing, we previously developed a PK-PD translational platform with bacterial dynamics that reliably predicted short-term monotherapy outcomes in Phase IIa trials from preclinical mouse studies. In this study, we extended our platform to include PK-PD models that account for drug-drug interactions in combination regimens and bacterial regrowth in our bacterial dynamics model to predict cure at the end of treatment and relapse 6 months post-treatment. The Phase III STAND trial testing a new regimen comprised of pretomanid (Pa), moxifloxacin (M), and pyrazinamide (Z) (PaMZ) was suspended after a separate ongoing trial (NC-005) suggested that adding bedaquiline (B) to the PaMZ regimen would improve efficacy. To forecast if the addition of B would, indeed, benefit the PaMZ regimen, we applied an extended translational platform to both regimens. We predicted currently available short- and long-term clinical data well for drug combinations related to BPaMZ. We predicted the addition of B to PaMZ to shorten treatment duration by 2 months and to have similar bacteriological success to standard HRZE treatment (considering only treatment success but not withdrawal from side effects and other adverse events), both at the end of treatment for treatment efficacy and 6 months after treatment has ended in relapse prevention. Using BPaMZ as a case study, we have demonstrated our translational platform can predict Phase II and III outcomes prior to actual trials, allowing us to better prioritize the regimens most likely to succeed.

## INTRODUCTION

Tuberculosis (TB) is a communicable disease caused by the bacillus *Mycobacterium tuberculosis* (*Mtb*). According to the World Health Organization (WHO), TB is a major cause of illness and the leading cause of infectious disease death worldwide (1). While therapies are available for TB treatment, the treatment is lengthy and burdensome, thus making it hard to eradicate (2, 3). Therefore, a huge emphasis has been placed on the need for more effective and shorter regimens (4, 5).

The development of novel drug combinations heavily relies on evidence from preclinical efficacy models. Among these, murine TB models are the most commonly employed (6, 7). Due to the highly evasive and resistant nature of *Mtb*, TB treatment is implemented as a cocktail of drugs rather than as monotherapy to decrease resistance and improve overall efficacy while dosing within safe ranges. New drugs are hence often evaluated in combination with a preexisting backbone of known drugs. Pretomanid (Pa) was tested with known TB drugs moxifloxacin (M) and pyrazinamide (Z) as a multidrug combination (PaMZ) in Phase III clinical trial STAND (NC-006) in 2015. However, STAND was halted early due to increased interest in BPaMZ, and analysis of its results showed 4-month PaMZ regimens failed to achieve non-inferiority to the 6-month regimen based on HRZE (the most common initial TB treatment regimen of isoniazid, H; rifampin, R; pyrazinamide, Z; and ethambutol, E) despite efficacy in preclinical combinations evaluated in mice (8). While the STAND trial might have been underpowered, it still highlights the complexity and difficulty of using early stage preclinical and clinical data to predict Phase III clinical endpoints, calling for a more robust translational platform to better inform later stage clinical outcomes.

Concurrent with the STAND trial, additional mouse efficacy studies suggested that adding the diarylquinoline bedaquiline (B) to PaMZ would increase efficacy (9) and the BPaMZ regimen achieved high rates of sputum culture conversion in participants with MDR-TB in the Phase IIb NC-005 trial (10). These results led to the Phase III SimpliciTB trial (NCT 03338621), designed to compare the efficacy and safety of a 4-month BPaMZ regimen to that of the 6-month HRZE-based standard of care in drug-susceptible TB. While SimpliciTB was underway, we expanded a previously developed translational pharmacokinetic-pharmacodynamic (PK-PD) platform that can predict Phase IIa outcomes based on preclinical mouse efficacy (11) to predict the efficacy results of the trial. The existing translational model incorporated bacterial dynamics into mouse exposure-response relationships and human PK profiles to predict Phase IIa outcomes for drugs tested as monotherapy and was validated on 10 1st and 2nd line TB drugs. The bacterial dynamics model detailed the interaction between bacterial growth and the mouse immune system prior to treatment, allowing us to account for these natural immune defenses too and not overpredict drug efficacy. Here, we extended this translational platform to predict the Phase IIb and III outcomes for drug efficacy in combination, using BPaMZ as a case study.

**Fig 1:**
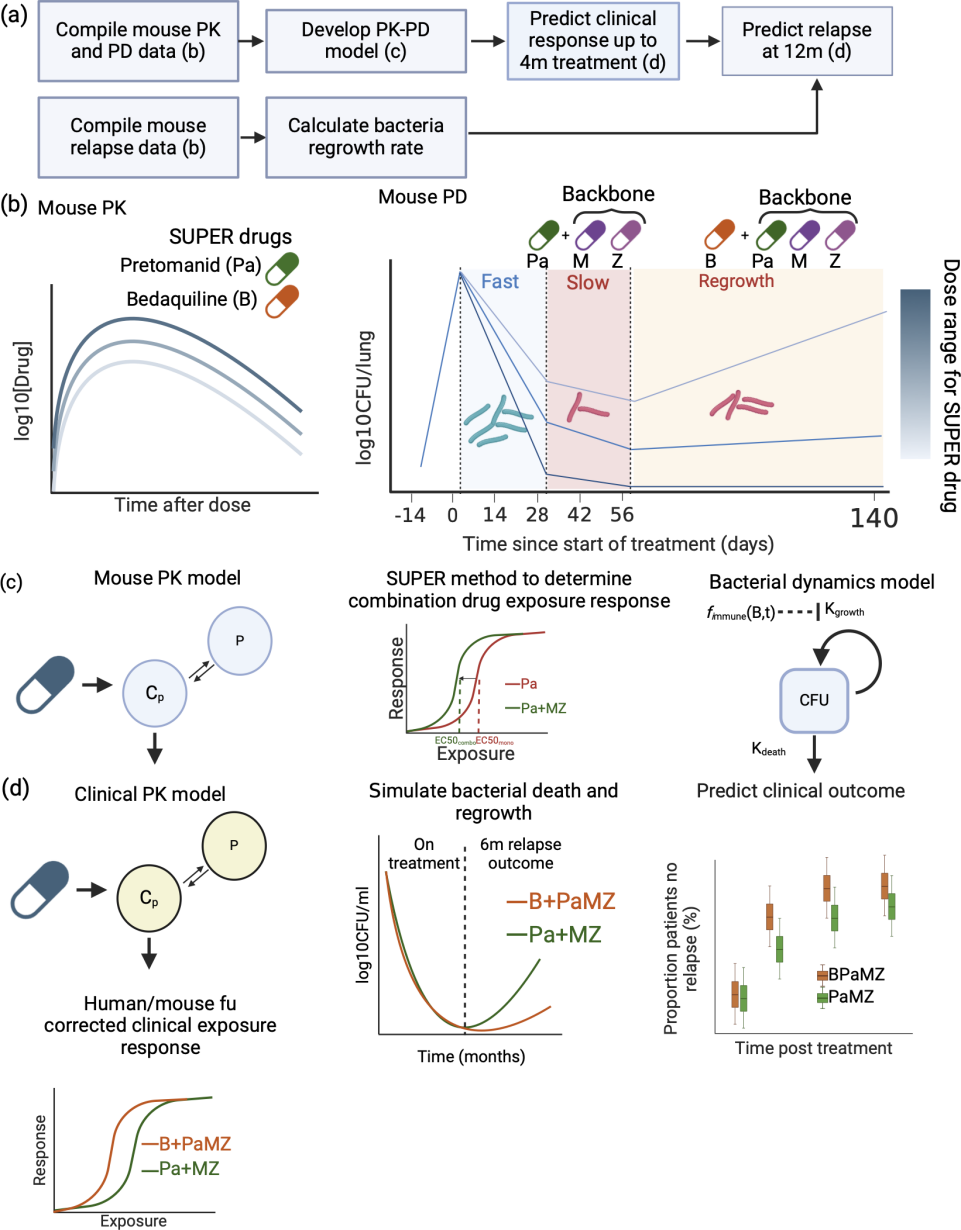
A three-step model to predict Phase IIb and III clinical outcomes using mouse PK-PD models. (**a**) Collect mouse PK data for the new drug of interest and mouse PD data of CFU over time for the drug as monotherapy, as well as both CFU and relapse data for drugs in combination with a known backbone regimen. (**b**) Build combination mouse PK-PD models using empiric method SUPER which assumes response is driven by the SUPER drug’s exposure, while backbone combination influences the response parameters of *E*_max_, EC50, and *γ*. (**c and d**) Simulate clinical outcomes using clinical PK models as well as translated exposure response relationship from mouse to human. Mouse EC50 is corrected using the ratio of drug fraction unbound in humans to that in mice.

While Phase IIa trials in TB tend to focus on early bactericidal activity of single drugs up to 2 weeks, Phase IIb trials typically study combination drug regimens over longer durations, measuring bactericidal activity up to 8 weeks. Phase III trials are the most stringent in assessing curative potential, measuring both culture status at the end of treatment, and the proportion of patients without relapse 12 or more months from the start of treatment. We, thus, added new components to extend our previous monotherapy translation platform: (i) pharmacodynamic drug-drug interactions included by measuring the effect of an established backbone regimen on the novel drug tested (referred to as SUPER since only a single “super” drug is tested against a backbone regimen) (12) and (ii) bacterial regrowth kinetics post-treatment (Fig. 1). The subsequent results demonstrate the utility of using preclinical mouse data to predict the sputum colony-forming unit (CFU) count over 8 weeks in Phase IIb, as well as the proportion of relapsing patients in Phase III (i.e., both short- and long-term efficacy outcomes) of a new drug regimen, an improvement from the original monotherapy Phase IIa prediction. Going forward, we expect to use this platform to prioritize the most effective drug combinations for evaluation in Phase IIb/III trials.

## RESULTS

### Comprehensive database of (B)PaMZ combinations in mice and humans

**Fig 2:**
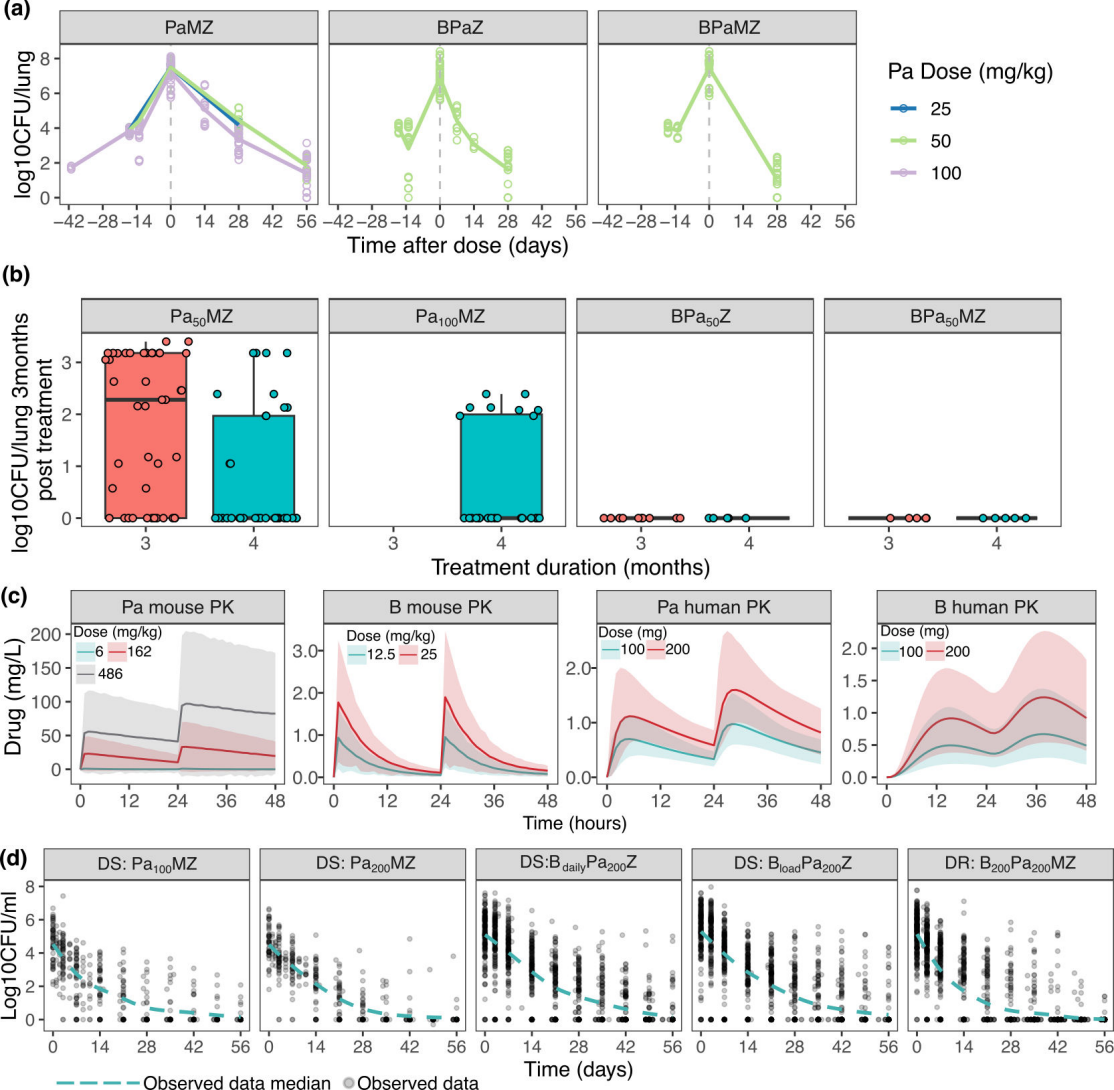
Observed data and model simulations used to train and validate the models. (**a**) Observed high-dose aerosol-infected mice inoculated with the drug-sensitive Mtb H37Rv strain prior to treatment with either PaMZ or BPaMZ. The only exception was PaMZ, where Pa 100 mg/kg treated mice were inoculated with low-dose aerosol infection instead. Pa doses ranged from 0 to 100 mg/kg, while other drugs were used at fixed doses: B 25 mg/kg, M 100 mg/kg, and Z 150 mg/kg. (**b**) Observed mouse relapse data 3 months after treatment ended. Treatment durations ranged from 3 to 4 months in mice. PaMZ, BPaZ, and BPaMZ relapse data were used for modeling regrowth. BPaM did not have sufficient relapse data for modeling regrowth and was hence not included in further clinical predictions. (**c**) Mouse and clinical PK simulations required for estimation of the exposure-response of the add-on SUPER drug to the backbone regimen. (**d**) Phase IIb observed clinical sputum count on treatment over a span of 8 weeks. The dashed line represents the mean CFU decline across all patients over time. Studies were NC-002 for Pa_100_MZ and Pa_200_MZ, and NC-005 for B_daily_Pa_200_Z, B_load_Pa_200_Z, and B_200_Pa_200_MZ. B was always given at 200 mg, except for the loading dose, which was administered as 400 mg for 14 days, followed by 200 mg thrice weekly until the end of treatment.

To build the translational model for the prediction of Phase IIb and III outcomes, preclinical BALB/c mouse PK (Table 1) and mouse PD data (Table 2) and clinical PK models (Table 3) were collected for model training and clinical observations, namely, sputum CFU count and first culture negative status for Phase IIb and relapse status 12 months from the start of treatment for Phase III trials were collected for model validation (Table 4). Monotherapy was first described using previously developed mouse PK-PD models describing the PK of Pa and B, as well as their efficacies as monotherapy. Drug combination efficacy describing PD in mice was collated from experiments performed at Johns Hopkins University (JHU) for drug regimens PaMZ, BPaZ, and BPaMZ (Fig. 2a). Additional mouse data on PaM and PaZ combinations were also collated and analyzed to understand the contribution of M and Z to Pa (Fig. S1). To better understand bacterial regrowth post-treatment, data from a relapsing BALB/c mouse model experiment performed at JHU were collected for treatment durations ranging from 1 to 4 months (9). Mouse CFU/lung was measured 3 months after treatment ended to measure the presence and extent of relapse for regimens Pa_50_MZ, Pa_100_MZ, BPa_50_Z, and BPa_50_MZ (Fig. 2b). Dosing information of other drugs is available in Table 2. BPaM analysis was excluded due to a lack of data for reliable estimates of bacterial regrowth post-treatment (*K*_net_) (Fig. S2). In total, we had 959 observations in mice for decline in CFU/lung and 125 observations for mouse relapse, including at the end of either 3 or 4 months of treatment, and 90 days after, allowing for robust testing of the translational framework. Mouse and clinical PK simulations were performed for the estimation of the exposure-response of the add-on SUPER drug to the backbone regimen (Fig. 2c and d).

**TABLE 1:** Mouse PK simulations

Regimen	Doses (mg/kg)	Data type	Data source
B	12.5, 25 single dose	PK simulation	Ernest et al. (11)
Pa	6, 9, 12, 18, 28.8, 50, 54, 162, 486 single dose; 100 daily for 4 or 8 weeks	PK simulation	Ernest et al. (11)

**TABLE 2:** Mouse PD data^*b*^

Regimen	Observations	SUPER drug	SUPER drug doses (mg/kg)	Data type	Data source	Treatment duration (days)
Initial						
Pa^*a*^	235	Pa	6.25, 10, 12.5, 25, 30, 50, 100, 200, 300, and 600	Mouse CFU/lung	JHU	14–63
PaM	84	Pa	50, 100	Mouse CFU/lung	JHU (9)	56
PaZ	92	Pa	50, 100	Mouse CFU/lung	JHU (9)	112
PaMZ	202	Pa	25, 50, 100	Mouse CFU/lung	JHU (9)	56
B^*a*^	75	B	12.5, 25,50	Mouse CFU/lung	JHU	79
BPaM	93	B	25	Mouse CFU/lung	JHU (13)	84
BPaZ	101	B	25	Mouse CFU/lung	JHU (13)	28
BPaMZ	77	B	25	Mouse CFU/lung	JHU (13)	28
Relapse						
PaMZ	100	Pa	50, 100	Mouse CFU/lung	JHU (9)	3, 4
BPaZ	15	B	25	Mouse CFU/lung	JHU (9)	3, 4
BPaMZ	10	B	25	Mouse CFU/lung	JHU (9)	3, 4

^*a*^ Data and PK-PD model have previously been described in Ernest et al. (11).

^*b*^ Doses of M and Z are 100 and 150 mg/kg, respectively.

**TABLE 3:** Clinical PK models used for simulations

Drugs	PK structure model	Study	Simulated doses	References
B	3-cmt model with transit absorption	NC-005 B_loading_	400 mg once daily for days 1–14, 200 mg three times per week for days 15–56	Svensson et al. (14)
		NC-005 B_daily_	200 mg once daily for 56 days	
		NC-008 B_[200/100]_	200 mg once daily for 56 days, then 100 mg once daily for 63 days	
Pa	1-cmt model with transit absorption and dose-dependent absorption, bioavailability, and volume	NC-002, NC-006	100 or 200 mg daily for 56 days or 119 days	Salinger et al. (15)

**TABLE 4:** Clinical PD observations from trials NC-002, NC-005, NC-006, and NC-008 used to validate the model^*a*^

Trial	Arm	Dosing	Time	Participants	Primary outcome	Ref.
NC-002	Arm 1: PaMZ in DS-TB	Arm 1: Pa 100 mg, M 400 mg, Z 1,500 mg daily	8 weeks	14	Sputum count CFU/mL from days 0 to 56	NCT01691534
	Arm 2: PaMZ in DS-TB	Arm 2: Pa 100 mg, M 400 mg, Z 1,500 mg daily				
NC-005	Arm 1: BPaZ in DS-TB	Arm 1: B 400 mg daily for 14 days followed by 200 mg three times a week. Pa 200 mg and Z 1,500 mg daily	8 weeks	148	Sputum count CFU/mL from days 0 to 56	NCT01498419
	Arm 2: BPaZ in DS-TB	Arm 2: B 200 mg daily. Pa 200 mg and Z 1,500 mg are dosed daily				
	Arm 3: BPaMZ in MDR-TB	Arm 3: B 200 mg, Pa 200 mg, M 400 mg, and Z 1,500 mg daily				
NC-006 (STAND-TB)	Arm 1: PaMZ in DS-TB	Arm 1: Pa 100 mg, M 400 mg, Z 1,500 mg daily	17 weeks	179	Culture negative status at 4 months and relapse at month 12	NCT02193776
	Arm 2: PaMZ in DS-TB	Arm 2: Pa 200 mg, M 400 mg, Z 1,500 mg daily				
NC-008 (SimpliciTB)	Arm 1: BPaMZ in DS-TB	B 200 mg (first 8 weeks) followed by 100 mg (9–17 weeks). Pa 200mg, M 400 mg, Z 1,500 mg daily	Arm 1: 17 weeks; Arm 3: 26 weeks	203	Relapse at month 12	NCT02342886

^*a*^ DS-TB, drug sensitive TB; MDR-TB, multidrug-resistant TB.

To validate the translational model predicted clinical outcomes, Phase IIb human sputum data over 8 weeks of treatment was collated from studies NC-002 (PaMZ) and NC-005 (BPaZ and BPaMZ) (Fig. 2d) (8, 10). Clinical data were collected from the STAND trial (NC-006) reporting adjusted proportions of favorable outcomes 12 months from treatment initiation as 74.5% and 85.2% on 4 months of Pa_100_MZ and Pa_200_MZ, respectively (8), and 97.9% for B_[200/100]_PaMZ in the SimpliciTB trial (16).

### A single-drug potency was sufficient to describe most mouse CFU profiles

In comparison to monotherapy, understanding the contribution of each individual drug to a regimen’s overall efficacy makes combination therapy a more complex problem to tackle. In preclinical settings, *in vitro* checkerboard analysis is commonly used to systematically evaluate drug efficacy of combinations (17, 18). Carrying out such a large range of doses in animals, however, is impractical due to the scale and number of combinations required. In animal studies, the new drug added to a regimen is often tested as a dose range, while the other drugs in regimen (e.g., MZ) are fixed, rather than testing all possible dose ranges for a four-drug combination. Therefore, we employed an empirical approach (SUPER) to quantify pharmacodynamic drug-drug interactions with only the exposure-response of a novel drug (either Pa or B) against a fixed dose backbone regimen (e.g., MZ or PaMZ) (12). Initially, we tested a more mechanistic model to account for the slower kill of persister bacteria by estimating both fast (initial 28 days of treatment) and slow-replicating (after 28 days of treatment) bacteria kill (19) as EC50_fast_ and EC50_slow_. We additionally tested a simpler model that assumed a homogeneous bacterial population as EC50. For B-containing regimens, no monotherapy data beyond 28 days was available, and a single EC50 value was estimated. With the PaMZ regimen, a single EC50 described the mouse CFU profile much better than when EC50_fast_ and EC50_slow_ were estimated, suggesting that MZ has good sterilizing potential against persisters (20–22). Similarly, a single EC50 described both PaZ and PaM regimens well (Fig. S1). These findings justified the use of a simpler model that assumes a homogenous bacterial population despite the extended treatment times beyond 28 days in our data set.

### SUPER model predictions overlapped well with observed sputum data over the course of treatment

**Fig 3:**
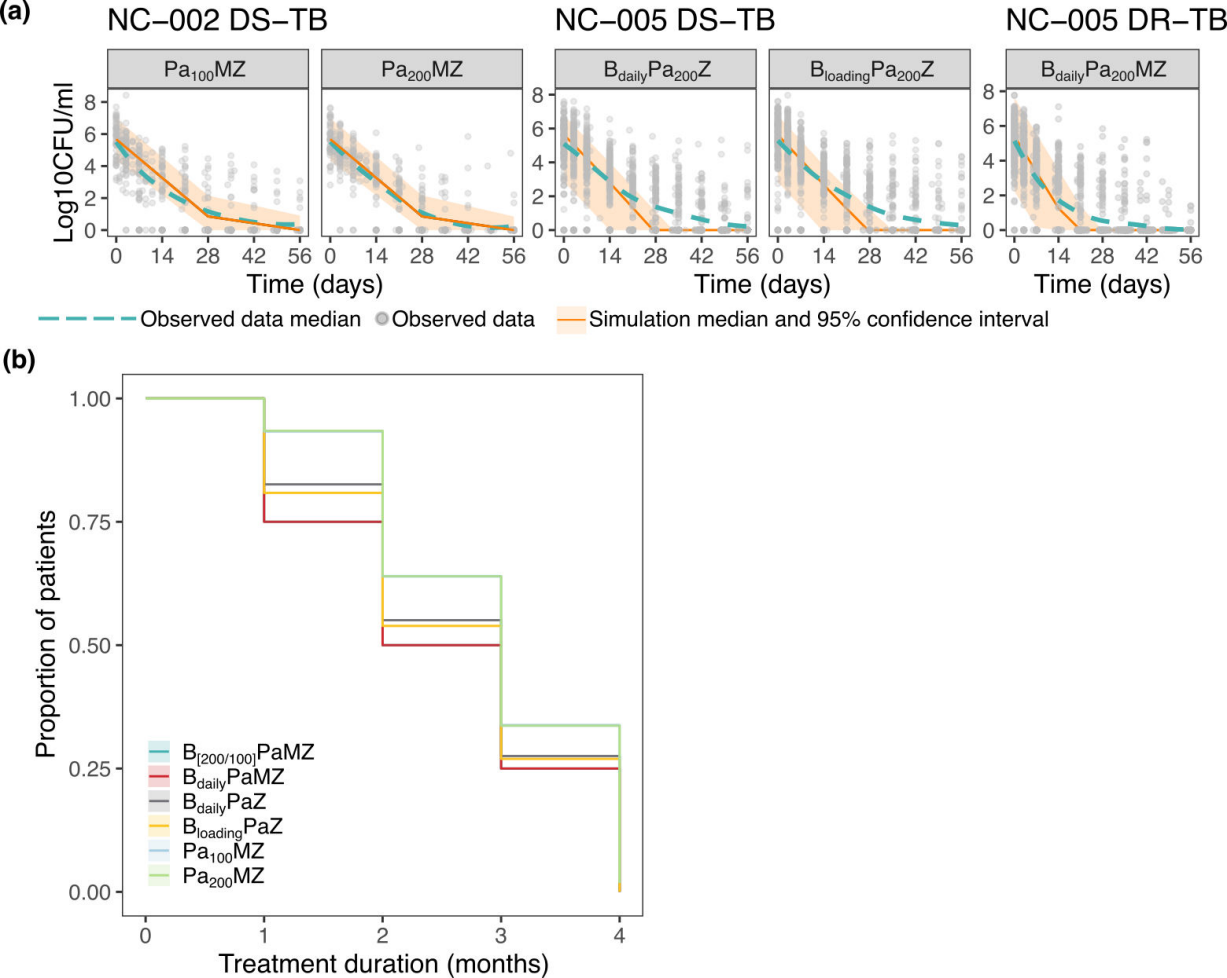
Phase IIb predictions at 8 weeks and 4 months of treatment. (**a**) Predicted bactericidal activity of the different drug regimens against clinical sputum CFU count over time. B_daily_ refers to B 200 mg given daily. B_[200/100]_ refers to B 200 mg daily for 8 weeks, followed by 100 mg daily. B_loading_ refers to a loading dose of B 400 mg daily for 2 weeks, followed by B 200 mg three times a week. Solid line represents median of model prediction, and dashed line represents mean sputum count of clinical data. (**b**) Kaplan Meier plot of the proportion of patients achieving first culture negative status across treatment duration. Further predictions to 4 months of treatment suggest that all regimens will convert have success bringing all patients to culture negative status at the end of treatment.

Using the exposure-response relationships derived from the preclinical mouse models and correcting for the fraction unbound between mouse and human for EC50 estimates, we were able to accurately predict the clinical 8 week sputum CFU count in NC-002 for Pa_100_MZ and Pa_200_MZ regimens using the SUPER approach. Our translated model prediction intervals fit well with observed clinical data, with our median prediction closely aligning with the observed median CFU drop over treatment duration (Fig. 3a). This prediction was accurate despite not quantifying the individual contributions of the backbone drugs, indicating that the SUPER method was sufficient to characterize a combined drug regimen’s exposure-response relationship for further translation. For NC-005 regimens BPaZ and BPaMZ, our models showed overprediction after approximately 21 days of treatment (Fig. 3a).

Upon extending the clinical simulation to 4 months, B-containing regimens were predicted to have more patients with culture-negative status at all treatment durations compared to PaMZ regimens. Adding M to the BPaZ regimen further improved this proportion of culture negative patients (Fig. 3b). B_daily_PaMZ, a daily dose of B 200 mg, and B_[200/100]_PaMZ, 8 weeks of B 200 mg followed by 8 weeks of B 100 mg, predictions were highly similar and overlapped. Similarly, Pa_100_MZ and Pa_200_MZ regimens had no significant differences in the proportion of culture-negative patients (Fig. 3b). At the end of 4 months, all regimens were predicted to achieve culture-negative status in their patient populations.

### Phase III clinical relapse outcomes can be predicted from bacterial regrowth in relapsing mouse models

As bacterial regrowth data after 3 months of treatment was sparse, we were not able to train a full bacterial dynamics model with confidence for regrowth. We, thus, used *K*_net_ as a composite measure of both bacterial growth and death instead to describe the net bacteria regrowth. In comparison to non-B-containing regimens, *K*_net_ was much slower with B onboard (Table 5). This could be a result of the sterilizing activity (9) and long half-life (23) of B. Moreover, in B-containing regimens, with Z onboard, *K*_net_ was even smaller, indicating B in combination with Z had even better sterilizing activity. In estimating relapse, we found that the spread in *K*_net_ quantified using between subject variance in mice was also an important factor in predicting the proportion relapsing. A larger spread of *K*_net_ values indicated a higher probability of relapse, even when median *K*_net_ was very close to 0. At the end of 4 months of treatment in mice, PaMZ regimens had a variance of 0.543, which was similar in both Pa_50_MZ and Pa_100_MZ regimens, while BPaZ and BPaMZ both had variance close to 0 as no mouse relapsed with 3–4 months of treatment (Fig. 2b).

**TABLE 5:** Mouse PK/PD models parameter in fast replicating, slow replicating bacteria, and relapse phase using monotherapy and combinations^*a*^

Drug regimen	SUPER drug	Parameter	Value	Relative standard error	Unit	SUPER drug fraction unbound ratio of human vs mouse
B	B	*E*_max_	0.515	0.007	day^−1^	1.0 (24)
		EC50	0.228	0.0027	mg/L	
BPaZ	B	EC50	0.151	0.008	mg/L	
		*E*_max_	0.515	FIX	day^−1^	
		*K*_net_	0.0044	1.43	day^−1^	
BPaM	B	EC50	0.7928	0.00005	mg/L	
		*E*_max_	0.515	FIX	day^−1^	
		*K*_net_	0.0126	1.52	day^−1^	
BPaMZ	B	EC50	0.00000226	0.0324	mg/L	
		*E*_max_	0.690	0.0208	day^−1^	
		*K*_net_	0.0052	0.930	day^−1^	
Pa	Pa	EC50	3.46	0.00306	mg/L	0.71 (25, 26)
		*E*_max_	0.429	0.00122	day^−1^	
		*γ*	0.374	0.0121		
PaZ	Pa	EC50	0.0765	0.0033	mg/L	
		*E*_max_	0.604	0.00666	day^−1^	
PaM	Pa	EC50	0.526	0.000108	mg/L	
		*E*_max_	0.429	FIX	day^−1^	
		*γ*	2.309	0.00007		
PaMZ	Pa	*E*_max_	0.406	0.000411	day^−1^	
		EC50	0.0724	0.000820	mg/L	
		*K*_net_	0.0492	0.366	day^−1^	

^*a*^ *E*_max_, maximum rate of bacterial kill per day; EC50, the concentration of drug required to reach 50% of the maximum rate of bacterial kill per day; *K*_net_, the net growth rate of bacterial after treatment has ended; *γ*, the Hill coefficient determining the steepness of the exposure response curve.

**Fig 4:**
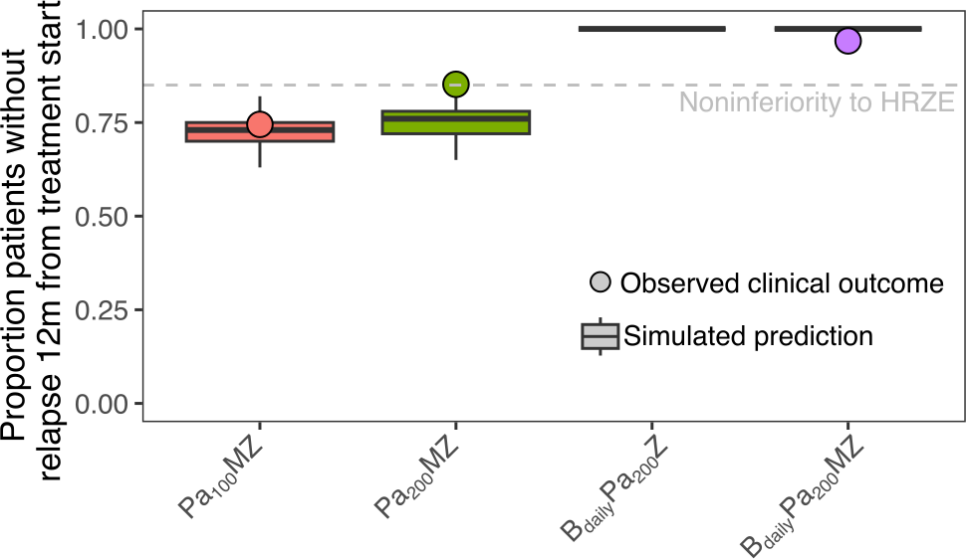
Regimen rank-ordering based on the proportion of patients without relapse 12 months since the start of treatment. Box plots represent the 95% CI, the circles observed proportion from STAND TB, and the dashed line the percent noninferiority to HRZE. Our simulations suggest that 4 months of either PaMZ regimen is inferior to B-containing regimens. Among the B-containing regimens, however, all regimens do similarly well, regardless of the dosing schedule. B_daily_Pa_200_Z did not have an observed clinical outcome.

The primary efficacy outcome of many Phase III TB trials is the proportion of patients with an unfavorable outcome 12 months after treatment initiation, where unfavorable status may be due to failure to achieve culture negativity on treatment, or recurrent positivity, i.e., relapse, after achieving negativity, or other causes (8, 16). The focus here was on bacteriologically relevant events. *K*_net_ from relapsing mouse models (Fig. S2) was, thus, used to simulate bacterial regrowth post-treatment for an additional 8 months following 4 months of treatment. Relapse was defined as CFU ≥ 1, the lower limit of bacteria detectable via culture. Each study was simulated according to its own baseline demographic information. The proportion of patients with undetectable CFU (no relapse) was predicted well in PaMZ regimens (Fig. 4) with the actual clinical trial adjusted proportions of unfavorable outcomes (74.5% Pa_100_MZ observed proportion, 85.2% Pa_200_MZ observed proportion) being close to our model predictions [73% (63%–80.5%, 95% confidence interval (CI)] Pa_100_MZ, 76% [(67%–82.5%, 95% CI) Pa_200_MZ]. Similarly, the efficacy of BPaMZ was also captured with 99% relapse-free survival in our relapse model, which was close to the 96.8% bacteriological success observed in SimpliciTB, thus predicting BPaMZ to be equivalent to standard of care in terms of relapse despite the shortened treatment duration by 2 months. However, BPaMZ was not noninferior to standard to care in terms of overall rate of unfavorable outcome. Notably, as B has a very long half-life, we observed that B was still at efficacious levels 6 months post-treatment in humans, potentially explaining the low rate of relapse (Fig. S5). These results demonstrate the model’s value for the prediction of Phase IIb and Phase III outcomes that enable us to rank and prioritize regimens before they go into clinical trial.

### The SUPER method was sufficient to detail drug combination efficacy in mice and humans

We validated our models with the SUPER method to be robust and stable by using visual predictive checks (VPCs) of our model fits over multiple different PaMZ and BPaMZ regimens using Pa at different doses (Fig. S1). The models were all able to describe the mouse data adequately using the SUPER method, with all the models converging well and model confidence intervals overlapping well with the data.

To further understand the success of BPaMZ over PaMZ, we explored our model-derived clinical exposure-response of the SUPER drugs with various combinations of their drug backbone (Fig. S3). Adding Z to Pa had an overall additive effect, improving upon Pa potency as evidenced by a 45.2-fold decrease in EC50, and increasing *E*_max_ by 1.41-fold. The combination of Pa with M was less beneficial than the combination with Z, with PaM EC50 decreasing by 6.58-fold compared to Pa monotherapy. The PaM model fit did not benefit from re-estimating *E*_max_. Interestingly, the PaMZ regimen had a similar *E*_max_ to Pa monotherapy, but was of a similar potency to PaZ, with a 47.8-fold decrease in EC50 compared to Pa monotherapy (Table 5). The full exposure response curves are plotted in Fig. S3.

Interestingly, we observed antagonism when M was combined with B in BPaM, where B EC50 increased by 3.5-fold compared to monotherapy B EC50. BPaZ was also additive in nature, but its effect was not as pronounced as with PaZ, with B EC50 improving in potency with a 1.51-fold decrease. Interestingly, the greatest synergism between all four drugs occurred with the BPaMZ regimen, which had a notably higher *E*_max_ (1.34-fold increase) and lower B EC50 (>1,000-fold decrease) compared to all other B-containing regimens. While only PaMZ was able to reach *E*_max_ at clinically observed steady-state concentrations of Pa 200 mg daily, all B-containing regimens reached *E*_max_ at clinically observed steady-state concentrations with 200 mg B daily, with BPaMZ having an almost immediate exposure at *E*_max_. This highlights BPaMZ’s superiority over the other regimens. This exposure-response analysis, thus, highlights the utility of the SUPER method in understanding drug regimen efficacy *in vivo* despite not accounting for the individual contributions of each drug.

## DISCUSSION

We extended our previous translational platform for predicting short-term monotherapy clinical outcomes to include long-term combination treatment outcomes: bacterial burden and proportion of bacteriologically relevant unfavorable outcomes. Using preclinical mouse data of drug regimens, we trained PK-PD models to capture the overall combination efficacy of the regimen and predict clinical outcomes for Pa_100_MZ, Pa_200_MZ, and BPaMZ. This expanded platform was able to predict Phase IIb sputum CFU counts over 8 weeks, as well as long-term outcomes (proportion of patients with undetectable CFU at the end of 4 months of treatment and bacteriologically relevant unfavorable outcomes 8 months post-treatment) to help prioritize the most efficacious drug regimens for clinical testing.

To understand the efficacy of combination drug regimens, PD drug-drug interactions need to be accounted for to determine the overall drug combination’s efficacy. Translational methods such as the generalized pharmacodynamic interactions (GPDI) method have been developed to address this problem but require a rich data set of dose ranges for every single drug in 2-to-4-way combinations. This is achievable using *in vitro* dosing studies, where high-throughput screens can be carried out. We have previously found mouse studies to be the preferable model for preclinical to clinical translation, however, because the mouse immune system also contributes to treatment efficacy (19). As mouse models are more costly, it is not as practical to test a wide range of drug doses and combinations. Thus, we employed the SUPER method which only requires dose ranging information for the new drug, against an established backbone regimen where clinically efficacious doses have been previously determined (12).

Despite not knowing the individual contributions of each drug to the overall regimen efficacy, we found that we could still understand the overall regimen efficacy and impact of different backbones on the SUPER drug (Fig. S3). We were also able to predict clinical outcomes for both PaMZ and BPaMZ drug regimens using the SUPER method, showing that drug regimen predictions could be made using only a dose range for the novel drug while keeping fixed doses for the rest of the regimen. This saves both time and money spent on preclinical experiments to evaluate the overall efficacy of a drug regimen.

Our model predictions were validated mainly using visual predictive checks (VPC), a gold standard in the pharmacometrics field, done by overlaying our prediction with the observed clinical data (27). As we did not refit our model to observed clinical data and, thus, do not have model diagnostics, the VPC instead provides an unbiased visual display of the actual model’s fit. The VPC, thus, ensures our predictions are reliable.

After validating our methods, we assessed whether this translational framework could be used to rank and prioritize drug regimens. While regimens showed differences in the proportion of culture negative patients during treatment, all regimens had close to culture negative status at the end of 4 months of treatment. This suggests that time to first culture negativity might not be the most discriminative measure in evaluating regimen efficacy. The 12-month follow-up from start of treatment is, thus, highly critical in prioritizing regimens. With Phase III relapse outcomes, we observed and were able to accurately predict, PaMZ to be less efficacious, and BPaMZ to be more efficacious, than the standard of care. This suggests that the relapsing mouse model is useful for regimen development, as knowing *K*_net_ to model relapse is key in understanding a drug regimen’s potential for clinical success compared to other regimens.

Deriving the exposure-response relationship further helps us evaluate the appropriate dosing for the novel drug being tested (28). In the case of Pa, it is likely that TB patients may benefit from a dose higher than 200 mg where safety allows in order to increase efficacy and have higher drug exposure close to *E*_max_. B, on the other hand, is at optimal dosing due to its high accumulation with repeated dosing (Fig. S3). This, thus ,highlights how PK-PD modeling can help further evaluate a dose.

In our model, we assumed that all bacteria over the course of 4 months of treatment belonged to the fast-growing population. This is a reasonable assumption to make, as bacterial persisters, which are known to be among the slow-growing population of bacteria, tend to make up less than 1% of the overall bacterial population (29). We, thus, can logically attribute most of the drop in sputum CFU clinically to a fast-growing population of bacteria. The only exception, however, was B, which was predicted to have high early bactericidal activity in our Phase IIa predictions but did not show the same result clinically (11, 30). Overprediction of the clinical bactericidal activity of B-containing regimens over 56 days was also observed in the current study. The reasons for such overprediction are likely multifactorial but are presumed to include the slower attainment of steady-state exposures inside caseous TB lesions that are not well represented in BALB/c mice and the contribution of the active B metabolite (M2) in mice that is not currently accounted for in the translational platform. (11)

As the SUPER method was able to capture the overall efficacy of the drug regimen, we suggest performing a dose range for only the novel drug, alongside a fixed backbone of doses of the other drugs, for at least three or four doses for a reliable EC50 estimate. This proposed experimental design can save a huge number of animals and costs, while still being able to derive a translatable exposure-response relationship.

In our simulations, we have found that the rate of kill does not vary much between most presented baselines prior to treatment of 6–8 log_10_CFU commonly reported in clinical trials. This was important to test as patient baselines are often variable and hard to predict as we often do not know the timeframe between a patient’s TB infection and presentation at a clinic (11). For validation, we, thus, simulated from reported patient baseline CFUs to for easier visualization. Without knowing anything about a prior population, we recommend a reference baseline of 7 log_10_CFU instead.

When analyzing mouse relapse data, we realized that in experimental groups with high proportions of mouse relapse, the relapse growth rate was highly variable between the mice. On the other hand, in groups where there was little to no relapse, this variability was much lower. We, thus, hypothesized that we could use a similar distribution of the growth rates to predict relapse clinically and validated this with observed clinical data.

In summary, our translational platform is a robust tool that can effectively distinguish combination regimens based on clinically relevant outcomes. Using PK-PD models built from preclinical data to predict both sputum data in Phase IIb and relapse data in Phase III, we have demonstrated a workable framework that helps us prioritize the most promising regimens for actual trials. This can help us save costs with much less trial and error involved when testing novel regimens to eradicate TB for good.

### Limitations

While our model was able to predict Phase IIb outcomes with good precision for PaMZ, we overpredicted the sputum CFU decline for BPaZ and BPaMZ regimens. This was likely due to an interesting phenomenon where B has been observed to be more active in mice compared to clinical models, resulting in the translated model overpredicting the CFU drop despite correcting for the free fraction of drug unbound differences between mouse and human (11). We had similarly reported this when predicting Phase IIa outcomes using B as monotherapy (11). Future work will involve validating similar models in C3HeB/FeJ mice which form necrotic lesions in the lung, making the model more similar to human TB pathophysiology and have lower sensitivity to B despite using the same strain of bacteria (31).

Our model was not able to capture the clinical observation of Pa_200_MZ as effectively as we hoped, as the *K*_net_ values between Pa_200_MZ and Pa_100_MZ were rather similar. This could be due to insufficient doses to properly elucidate a proper exposure response relationship for relapse. However, we do note too that STAND-TB was underpowered as it was halted early, and thus, while the observed data are still useful for gauging model performance, we cannot reliably state the observed points as reflective of a larger trial population should STAND-TB have been fully completed.

Another limitation is that although our model could predict bacteriologically relevant clinical outcomes well, there are other contributing causes of unfavorable outcome. Favorable response does not only consist of the presence or absence of relapse. Safety aspects of the regimen, such as withdrawal due to adverse effects, also need to be considered. The failure of BPaMZ to demonstrate non-inferiority vs HRZE in SimpliciTB was largely due to withdrawals during treatment for adverse events (16).

In this study, we also relied only on the traditional marker for efficacy, bacterial CFU. Other promising biomarkers are being developed, such as RS ratio, which could potentially be used as an early biomarker analysis for activity, instead of relying on CFU, which has a minimum doubling time of 14.7 h (32), much longer than most other culturable bacteria. Further work on validating these biomarkers is ongoing.

### Conclusion

Here, we extended our translational platform (11) to include drug combinations and predict longer treatment duration outcomes in both Phase IIb and III. Using the SUPER method, we were able to account for pharmacodynamic drug interactions in a combination regimen in a cost-effective manner by performing dose ranging only for a single drug. Furthermore, we showed for the first time the utility of the relapsing mouse model for informing predictions of Phase III outcomes using our platform, highlighting that culture-negative status alone was insufficient to predict Phase III outcomes well. Despite having an overprediction of Phase IIb outcomes with BPaZ and BPaMZ, our relapse model was still able to accurately predict bacteriologically relevant outcomes in the BPaMZ trial. This demonstrates the usefulness of PK-PD models in helping to translate preclinical results to clinical outcomes, and prioritizing which regimens should be used for further testing.

## MATERIALS AND METHODS

### Database compilation

All data used in the translational platform development were summarized in Tables 1 and 2. Preclinical plasma concentrations were collected from B, Pa, M, and Z monotherapy. Mice lung CFU counts after treatment were collected from both monotherapy and various combinations (PaZ, PaM, BPaM, BPaZ, PaMZ, BPaMZ) as efficacy data. Clinical PK data of Pa and B were simulated using a published human population PK model. Sputum CFU counts for PaMZ, BPaZ, BPaMZ in Phase IIb trials were collected from published clinical studies, with individual CFU data from NC-002 and NC-005 obtained from TB Alliance via personal communication. Phase III outcomes for PaMZ and BPaMZ were collected from published sources (8, 16).

### Model development

All model development and simulation were performed using NONMEM (version 7.4) and Perl speaks NONMEM (PsN) using first-order conditional estimation with interaction (FOCE-I) as the estimation method. Model diagnostics, model validation, and data visualization were performed in R and R studio (version 4.1.3) using Xpose 4 and ggplot R packages. Mouse PK and PK-PD models were developed and optimized based on statistical (significant change in objective function value), graphical (goodness of fit plots), and simulation-based diagnostics (visual predictive checks).

### Mouse PK-PD model development

Mouse PK-PD models were developed based on our prior integrated mouse PK-PD model that describes bacteria growth, death, and adaptive immune effect without drug treatment (19) for the prediction of clinical Phase IIa outcomes for drugs as monotherapy. To account for PD drug-drug interactions in the model, an empirical approach (SUPER), measuring the total efficacy of the regimen using only the shifted exposure-response of the novel drug with the backbone regimen added. This was done by modeling only the exposure-response parameters described using an *E*_max_ model (*E*_max_, EC50, and *γ*) of the novel drug (either Pa or B) in combination with a fixed drug backbone with reference to the novel (SUPER) drug’s PK as the main driver of the drug combination’s response. This would allow us to determine the effect of the backbone on the SUPER drug by estimating the shift in EC50 when comparing EC50 estimated from both monotherapy and combination. Full method details are listed in the supplementary material.

### Relapsing mouse model

To account for relapse, a simplified baseline model measuring only the net growth rate constant (*K*_net_) of bacteria was used to describe mice relapse. *K*_net_ was chosen as only the bacterial growth in mouse lung, rather than real-time bacterial growth dynamics, could be quantified, making it difficult to quantify both a growth and death rate separately. Net growth of bacteria after treatment stopped was calculated. Treatment duration and Pa dose were also added as covariates to account for differences in experiments.

### Translational model for Phase IIb 8-week sputum count and Phase III 4-month treatment outcomes

To validate our model against existing trial results, the same reported end points of the trials, namely, sputum count and culture negative status were simulated. To simulate the Phase IIb sputum count, clinical PK simulations of either Pa or B were combined with a translated exposure-response model derived from the mouse PK-PD models with the SUPER method to predict sputum CFU/mL. Translation from mouse to clinical was done by correcting the EC50 using the ratio of the fraction unbound drug in human vs mouse. Clinical model simulations of sputum count were carried out over a full period of 8 weeks using matching baseline demographics to patient populations from NC-002 (Pa_100_MZ, Pa_200_MZ) and NC-005 (B_daily_PaZ, B_load_PaZ, B_daily_PaMZ). In B-containing regimens, Pa 200 mg was used. Actual trial demographics were used to simulate variability in the patient PK profile and baseline CFU. To evaluate model predictions, the 95% prediction interval and the median line were overlapped with observed clinical data. Doses of M and Z were fixed at 400 mg and 1500 mg once daily, respectively.

Similarly, to predict culture status at the end of treatment, the clinical simulations of sputum count were extended to 4 months. One thousand simulations were conducted for each regimen. A culture-negative individual was defined as having a predicted CFU < 1, with a CFU count of 1 being the minimum amount of bacteria required for a positive culture result. The total predicted proportion of patients with negative sputum counts was then compared against the observed proportion of patients with negative sputum counts from NC-006 to validate the model predictions.

### Translational predictions of Phase III relapse outcomes

Clinical relapse outcomes were predicted by simulating the whole course of the trial, from 4 months of treatment to 8 months of bacterial regrowth after treatment had ended, giving a total duration of 12 months since treatment start. Clinical bacterial regrowth was simulated using the *K*_net_ and standard deviation derived from relapsing mouse models. Patients with no relapse were defined as having a predicted CFU < 1, with a CFU of 1 being the minimum amount of bacteria required to detect relapse. One thousand simulations were conducted for each regimen (Pa_100_MZ, Pa_200_MZ, B_daily_Pa_200_Z, B_daily_Pa_200_MZ). The proportion of predicted non-relapsed patients was then compared against the adjusted proportion of non-relapsed patients from NC-006 and SimpliciTB trials with favorable outcomes, defined as those with favorable outcomes out of an adjusted assessable total that removed nonviolent or accidental deaths, withdrawals due to adverse events, withdrawals due to investigator or participant decisions, non-adherence to study protocol, and loss to follow-up from the total assessable per the studies’ definitions of Modified Intent to Treat (8, 16).

## SUPPLEMENTAL MATERIAL

Supplemental figures aac.00615-24-s0001.docx Figures S1 to S5.

ASM does not own the copyrights to Supplemental Material that may be linked to, or accessed through, an article. The authors have granted ASM a non-exclusive, world-wide license to publish the Supplemental Material files. Please contact the corresponding author directly for reuse.
