# Supplementary material for: Prospectively predicting BPaMZ phase IIb/III trial outcomes using a translational mouse-to-human platform
Source: Antimicrob Agents Chemother. 2024 Sep 17;68(10):e00615-24. doi: 10.1128/aac.00615-24 (PMC11459968; doi:10.1128/aac.00615-24)
Supplement: Supplemental figures — Figures S1 to S5. [file aac.00615-24-s0001.docx]

Supplementary data and methods


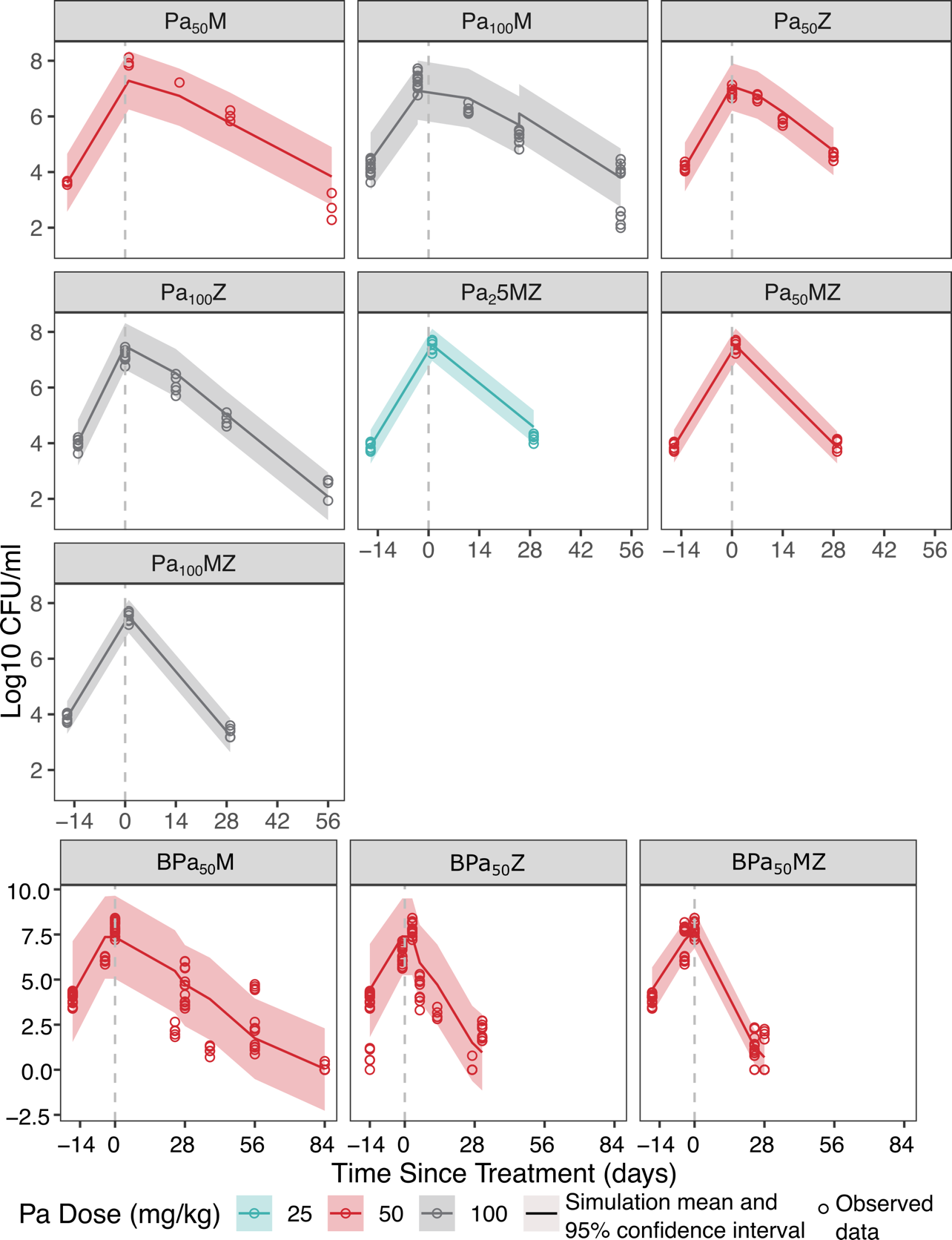


Figure S1. Visual predictive check of mouse CFU data over time with models trained using the SUPER method. Pa as SUPER drug in PaMZ: 2- and 3-way combinations are shown in the first 3 rows. Each color corresponds to their respective Pa dose. B as super drug in BPaMZ: 3- and 4-way combinations are in the bottom row, with each B-containing combination using a dose of Pa 50 mg/kg in mice. B was dosed at 25 mg/kg for all B-containing regimens.


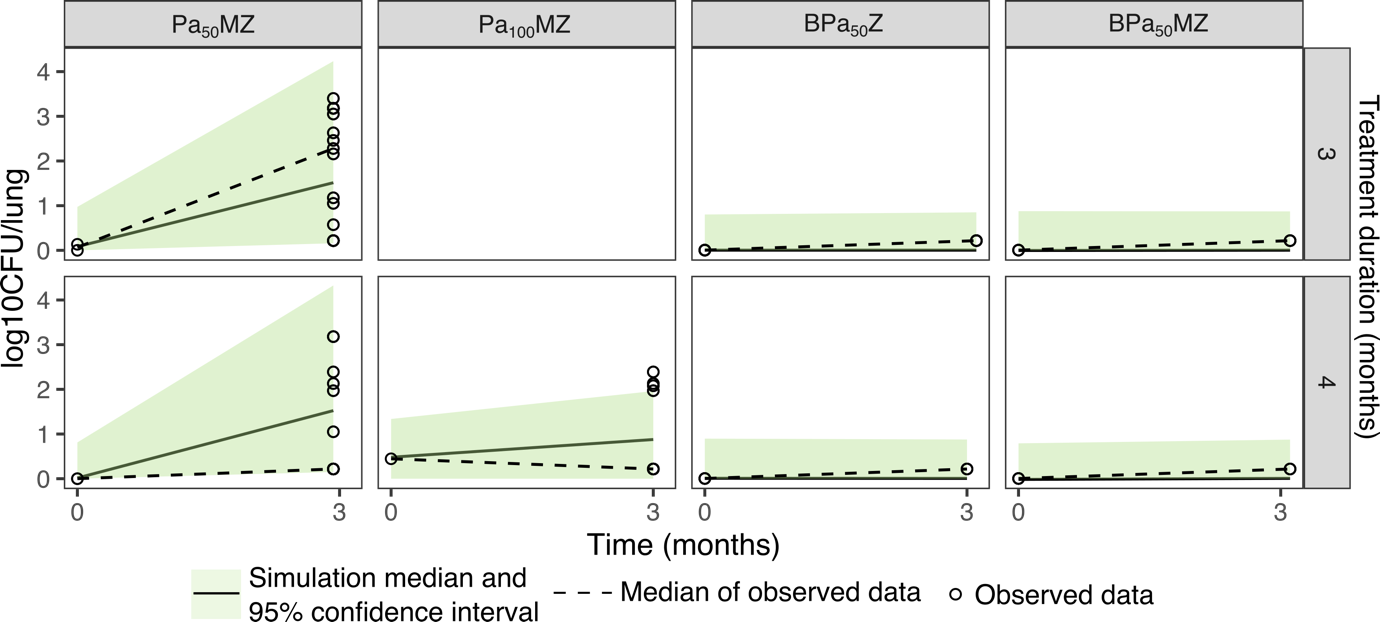


Figure S2. Visual predictive check for mouse relapse at different treatment durations. Ribbons represent the model prediction interval. The solid line represents the predicted median growth rate (KNET), and the dashed line, the observed median CFU at relapse. Month 0 represents the time at treatment end, while month 3 represents the end of incubation after treatment end and the time at which presence or absence of bacterial growth was assessed in the relapsing mouse model.


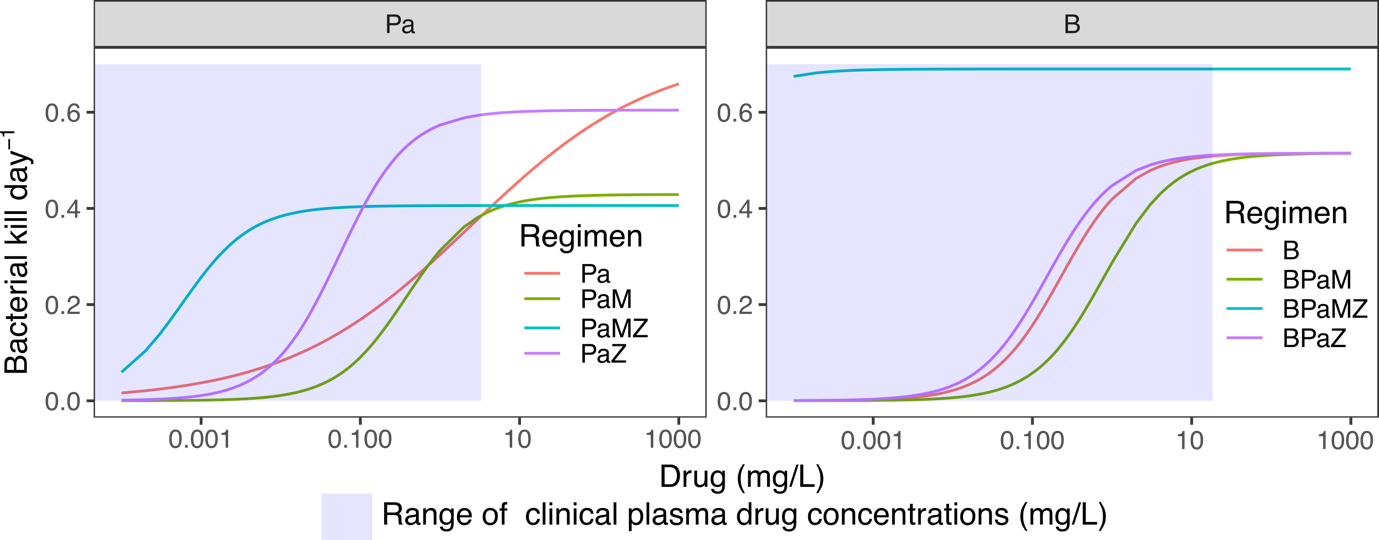


Figure S3. Exposure response relationships when either Pa or B was modelled as SUPER drug with different backbone regimens. If EC50_fast_ and EC50_slow_ were measured in the regimen, EC50_fast_ was used to be a fair comparison to the rest of the drugs that only had EC50_fast_. The clinical doses simulated were the final doses at 4 months of treatment using Pa 200 mg, and B 200 mg, as per the clinical regimens.

*Modeling the overall drug effect of a combination using the SUPER method*

*
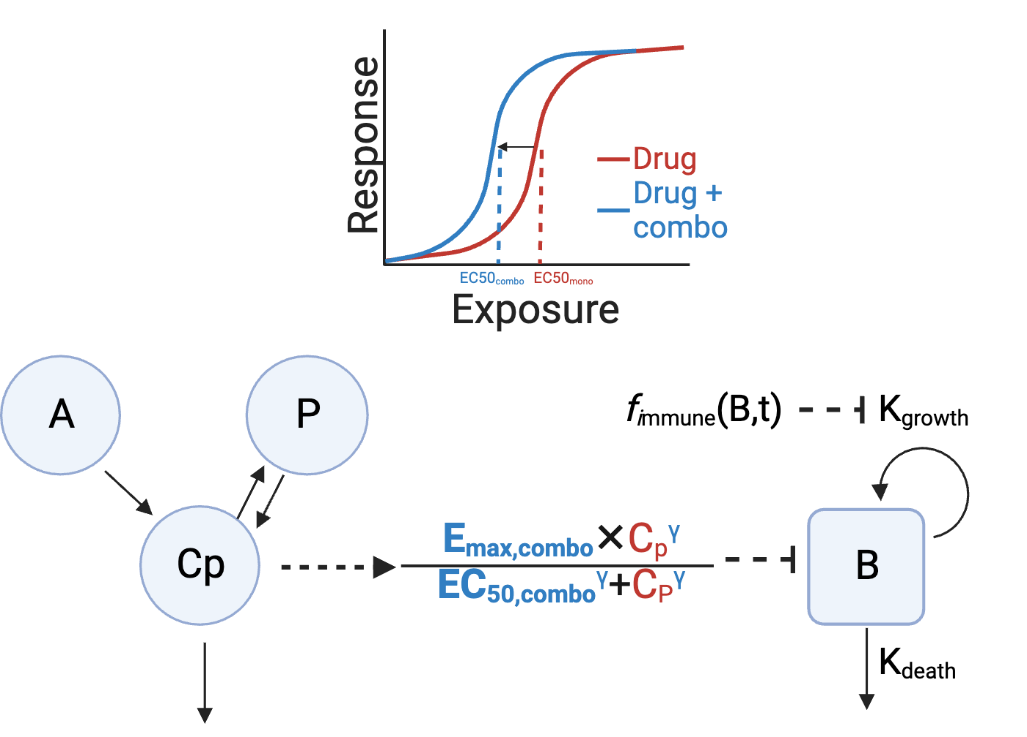
*

Figure S4. Schematic of an integrated PK-PD model accounting for the total drug effect combination using the SUPER method. In this method, we assume that response can be driven simply using the exposure of the SUPER drug alone using either the corresponding mouse or human PK simulation, and the addition of a backbone regimen shifts only the response parameters of E_max_ and EC50.


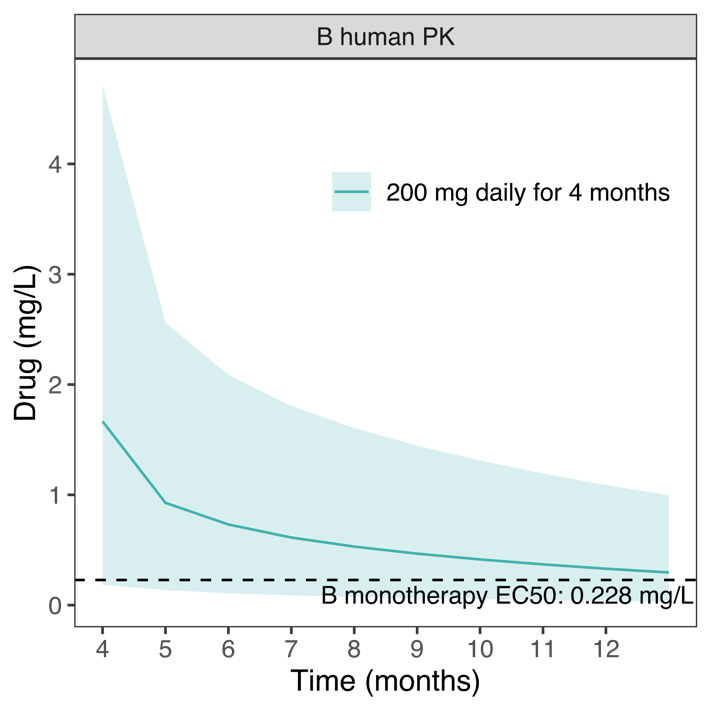


Figure S5. PK simulation of B 200mg daily 6 months after treatment discontinuation. Median B levels persist above B monotherapy EC50 for almost the entire duration of observation.

To model a combined drug regimen’s effect, the overall effect of the drug combination needs to be accounted for. However, as it is impractical for animal experiments to have a dose range for each drug in a 3 or 4 way combination, we decided to utilize an empiric method, SUPER, to model the drug response of the drug combination. SUPER assumes that response can be driven simply using the exposure of the SUPER drug alone and that the addition of a backbone regimen shifts only the response parameters of E_max_ and EC50. To account for pharmacokinetic drug-drug interactions that could change drug exposure of the SUPER drug, we ran all 4 drugs, (B, Pa, M and Z) through the Lexicomp drug interaction checker^1^ and found no significant drug interactions. We thus could safely assume that the PK of the SUPER drug would not change significantly when in combination with a backbone regimen.

Monotherapy mouse PK and PD was described using previously published integrated PK-PD models with bacterial dynamics from Ernest et al. for both Pa and B, the SUPER drugs of interest in this paper. To account for the addition of the drug backbone to the SUPER drug, we re-estimated the exposure-response parameters E_max_, EC_50_, and γ.

Similar to our previous published translation on monotherapy, we use the same bacterial dynamics model to account for mouse immune response ^2^ (Eq. 1). Drug effect (EFF) was defined as bacterial killing rate (day^-1^) and was defined as the exposure response of the SUPER drug with backbone effect (Eq. 2). All the effect models were assumed to be direct effect models. The observed output, bacterial number (B) was defined as the remaining bacteria after accounting for the dynamic processes of bacterial growth (K_g_), natural bacterial death (K_d_) and drug induced bacterial death (EFF) (Eq. 3).

$\frac{dB}{dt}=K_{g}\times B\times\left( 1-\frac{K_{B}\times B^{\gamma_{B}}}{B_{50}^{\gamma_{B}}+B^{\gamma_{B}}} \right)\times\left( 1-\frac{{K_{T}\times t}^{\gamma_{T}}}{{T_{50}}^{\gamma_{T}}{+t}^{\gamma_{T}}} \right) -K_{d}\times B Eq. 1$

${EFF}_{combo}=\frac{C_{P}^{\gamma,combo}\times E_{max,combo}}{{EC}_{50,combo}^{\gamma,combo}+C_{P}^{\gamma,combo}} Eq. 2$

$\frac{dB}{dt}=K_{g}\times B\times\left( 1-\frac{K_{B}\times B^{\gamma_{B}}}{B_{50}^{\gamma_{B}}+B^{\gamma_{B}}} \right)\times\left( 1-\frac{{K_{T}\times t}^{\gamma_{T}}}{{T_{50}}^{\gamma_{T}}{+t}^{\gamma_{T}}} \right) -K_{d}\times B-{EFF}_{combo}\times B Eq. 3$

$B$*: bacterial number*

$t$*: incubation time since inoculation*

$K_{g}$*: bacterial growth rate*

$K_{d}$*: bacterial natural death rate*

$K_{B}$*: bacterial number-dependent maximal adaptive immune effect*

$B_{50}$*: bacterial number that results in half of* $K_{B}$

$\gamma_{B}$*: steepness of bacterial number-dependent immune effect relationship*

$K_{T}$*: incubation time-dependent maximal adaptive immune effect*

$T_{50}$*: bacterial number that results half of* $K_{T}$

$\gamma_{T}$*: steepness of time-dependent immune effect relationship*

*EFF_combo_: bacterial killing rate*

*E_max,combo_: the maximal level of drug A effect*

*EC_50,combo_: the plasma concentration of SUPER drug that results in half of the maximal drug combination effect*

*C_p,SUPER_: the plasma concentration of SUPER drug*

*γ_combo_: the steepness of the relationship between the plasma concentration and drug combination effect*

To account for a slower kill rate after 28 days where mouse CFU data was available, EC50_fast,combo_ was estimated to account for bacterial kill ≤28 days, and EC50_slow,combo_ for bacterial kill > 28 days in place of EC50_combo_.

*Bacterial regrowth after treatment end*

Bacterial regrowth post treatment was described as K_net_, the net rate constant of bacteria change over time (Eq. 4). K_net_ is considered to be a result of bacteria growth, death and adaptive immune effect post-treatment. However, while B was completely cleared from mice within 2 weeks post 4 months of daily gavage treatment, B had a notably long half-life in humans, with B having effective plasma concentrations 6 months after treatment. Hence we decided to account for this using EFF_mono_, to account for the monotherapy drug effect by B after treatment had ceased (Eq. 5 and 6). With Pa, EFF_mono_ was assumed to be 0 as it was cleared out by the body at a faster rate.

$\frac{dB}{dt}=Knet \times B$Eq.4

${EFF}_{mono}=\frac{C_{P}^{\gamma,mono}\times E_{max,mono}}{{EC}_{50,mono}^{\gamma,mono}+C_{P}^{\gamma,mono}} Eq. 5$

$\frac{dB}{dt}=Knet \times B-{EFF}_{mono}\times B$Eq.6

$K_{net}$*: bacterial net growth rate*

*EFF_mono_: bacterial killing rate on B as monotherapy*

*E_max,mono_: the maximal level of B effect*

*EC_50,mono_: the plasma concentration of B that results in half of the maximal drug effect*

*C_p:_ the plasma concentration of B after treatment end*

*γ_mono_: the steepness of the relationship between the plasma concentration and drug effect*

1. Lexicomp® Drug Interactions - UpToDate. https://www.uptodate.com/drug-interactions/?source=responsive_home.

2. Zhang, N. *et al.* Mechanistic Modeling of Mycobacterium tuberculosis Infection in Murine Models for Drug and Vaccine Efficacy Studies. *Antimicrob. Agents Chemother.* **64**, (2020).
